# *Helicobacter pylori*-Related Metabolic Parameters and Premalignant Gastric Mucosa Histological Lesions in Swiss Bariatric Patients

**DOI:** 10.3390/microorganisms9071361

**Published:** 2021-06-23

**Authors:** Michael Doulberis, Noah Thierry Pierre, Giulia Manzini, Apostolis Papaefthymiou, Jannis Kountouras, Jolanta Klukowska-Rötzler, Stergios A. Polyzos, Simone Srivastava, Aristomenis K. Exadaktylos, Jürg Knuchel, Thomas Kuntzen, David S. Srivastava

**Affiliations:** 1 Emergency Department, University Hospital Inselspital of Bern, 3010 Bern, Switzerland; osagyefo@gmx.de (N.T.P.); jolanta.klukowska-roetzler@insel.ch (J.K.-R.); Aristomenis.Exadaktylos@insel.ch (A.K.E.); david.srivastava@hirslanden.ch (D.S.S.); 2 Division of Gastroenterology and Hepatology, Medical University Department, Kantonsspital Aarau, 5001 Aarau, Switzerland; juerg.knuchel@ksa.ch (J.K.); thomas.kuntzen@ksa.ch (T.K.); 3 Second Medical Clinic, School of Medicine, Aristotle University of Thessaloniki, Ippokration Hospital, 54642 Thessaloniki, Macedonia, Greece; appapaef@hotmail.com (A.P.); jannis@auth.gr (J.K.); 4 First Laboratory of Pharmacology, Aristotle University of Thessaloniki, 54124 Thessaloniki, Macedonia, Greece; spolyzos@auth.gr; 5 Private Practice, 4704 Niederbipp, Switzerland; 6 Department of General and Visceral Surgery, Kantonsspital Aarau, 5001 Aarau, Switzerland; giulia.manzini@ksa.ch; 7 Department of Gastroenterology, University Hospital of Larisa, Mezourlo, 41110 Larisa, Thessaly, Greece; 8 Gastroenterologie Thun—Bauch im Zentrum, 3600 Thun, Switzerland; srivastava@gmx.de; 9 Department of General Internal Medicine, Kliniken Hirslanden Beau-Site, 3013 Bern, Switzerland

**Keywords:** bariatric, *Helicobacter pylori*, *Hp*, metabolic syndrome, MetS, gastric atrophy, GA, intestinal metaplasia, IME

## Abstract

Obesity, as a major risk factor of metabolic syndrome (MetS), represents a pandemic, especially in Western societies, and is considered a risk factor for malignancies. *Helicobacter pylori* (*Hp*), is a definite carcinogen with global distribution. We aimed to investigate, for the first time in Switzerland, the main gastric mucosa premalignant histological lesions of bariatric patients in correlation with MetS components and *Hp* Infection (*Hp*-I). By reviewing retrospectively 94304 patient cases, a total of 116 eligible patients having undergone bariatric surgery were identified. The mean patient age was 48.66 years. *Hp*(+) patients were 24% (28/116). Presence of gastric mucosa atrophy was documented in 8/28 *Hp*(+) patients (29%) and (2/88) *Hp*(−) ones (2%) (*p* = 0.006). Gastric mucosa intestinal metaplasia was observed in 14/28 (50%) *Hp*(+) patients versus 3/88 (3.4%) of *Hp*(-) group (*p* < 0.0001). *Hp*(+) patients exhibited statistically higher arterial hypertension (*p* = 0.033). The homeostatic model of assessment insulin resistance was also statistically significantly higher for the *Hp*(+) group (*p* < 0.001). In a multivariate analysis, including arterial hypertension, gastric mucosa atrophy, and intestinal metaplasia as variables, statistical significance remained only for intestinal metaplasia (*p* = 0.001). In conclusion, *Hp*-I is associated with premalignant gastric mucosa histologic lesions and MetS components, including arterial hypertension and IR. Further large-scale prospective studies are required to confirm these findings.

## 1. Introduction

Obesity has reached pandemic dimensions nowadays, with more than 1.5 billion individuals, i.e., about one-third of the world’s population, regarded as obese or overweight [1]. Visceral adiposity, arterial hypertension, dyslipidemia, and type 2 diabetes mellitus (T2DM) with its accompanied insulin resistance (IR) are core components of metabolic syndrome (MetS) and are, apart from other comorbidities, cardio–cerebrovascular risk factors with devastating consequences [1,2,3]. Obesity develops due to a perpetuated disproportional energy intake and expenditure [1]. Beyond the aforementioned pathologies, obesity has been associated with an elevated risk of various cancers including upper and lower gastrointestinal tract malignancies [4,5]. In this respect, *Helicobacter pylori (Hp)*, a Gram-negative microaerophilic bacterium that colonizes primarily the stomach of more than 50% of people of the world has been characterized by the World Health Organization since 1994 as a class I (definitive) carcinogen [6,7]. *Hp* has been well established to cause gastritis [8,9], and up to 3% of the infected individuals develop gastric cancer (GC) and/or MALT lymphoma [10]. Specifically, *Hp*-infection (*Hp*-I) is causally related to the development of gastric mucosa atrophic gastritis (AG) and intestinal metaplasia (IM), as well as GC [11,12]. AG and IM with dysplasia are the main premalignant lesions detectable in most of the GCs [11,12,13]. Moreover, *Hp*-I has been repeatedly associated with MetS and IR, as well as its hepatic component nonalcoholic fatty liver disease, recently renamed as the metabolic dysfunction-associated fatty liver disease [14,15,16]. In this regard, the incidence of *Hp*-I in bariatric patients and characterization of the mentioned main histopathologic precancerous lesions being found incidentally in the gastric biopsies is an attractive correlation, which has been performed only in few studies [4,17,18,19,20,21,22,23,24,25,26]. In this retrospective study, we aimed to investigate, for the first time in Switzerland, the presence of the mentioned main histological precancerous lesions in gastric mucosa biopsies obtained in terms of a planned bariatric surgery and correlate them with a variety of metabolic parameters through the prism of *Hp*-I.

## 2. Materials and Methods

### 2.1. Study Design

This is a retrospectively designed study, which was conducted by implementing an electronic database of the academic tertiary general hospital Inselspital emergency department (ED) in Bern, Switzerland, for the timeframe of 1 January 2017–30 November 2018. The total amount of recorded isolated patient cases was 94,304. It is of note that Inselspital offers its medical services to around 2,000,000 people.

### 2.2. Ethical Considerations

The study complied with the last revision of the Declaration of Helsinki principles [27] and with the Guidelines of Good Clinical Practice [28]. Ethical approval was obtained by the cantonal (district) ethics committee in Berne, (Kantonale Ethikkommission Bern, Ref. No. KEK-BE: 010/2016). Since patients were fully anonymized prior to analysis, according to Swiss law, no informed consent was necessary.

### 2.3. Inclusion and Exclusion Criteria

Eligible were morbidly obese patients presenting at the ED of Inselspital in the above-mentioned time period, regardless of the claimed symptoms. Eligible cases were patients at least 16 years old or more and submitted to bariatric surgery. The patients’ exclusion criteria were as follows: missing significant data, such as histological findings; double-recorded cases; previous *Hp* eradication treatment; advanced malignancies diagnosed at post-bariatric surgery time; pregnancy and lactation; and use of antibiotics or PPIs the last four weeks prior to gastric biopsy.

### 2.4. Data Collection and Extraction

Morbidly obese patients undergoing an elective bariatric operation were recruited, as described below. *Hp*-I status was evaluated by the practical diagnostic “gold standard” histology from gastric mucosa biopsies [14,29,30], obtained either preoperatively (via a screening esophagogastroduodenoscopy) or intraoperatively. Histological diagnosis of *Hp* gastritis was confirmed by means of hematoxylin and eosin staining or a modified Giemsa/immunohistochemistry to better highlight/selectively label the *Hp* bacteria, respectively.

Patients’ records at the time of admission to the ED were stored in the clinical application “E.care” for Windows (E.care BVBA, ED 2.1.3.0, Turnhout, Belgium). “E.care” offers the advantage of instantaneous recall of medical reports, and other relevant data, while applying multiple filters of the “E.care” application. Patients’ records from ED were extracted to an Excel sheet (Microsoft^®^ Excel for Mac 2019, Microsoft Corporation, Redmond, WA, USA) with the utilization of multiple “E.care” filters. For the identification of the final eligible cases, we followed the following algorithm: Once data were extracted into an Excel sheet, we performed an electronic query of the total database (*n* = 94,394 ED cases) by searching into “diagnosis list” column with the keywords: “bariatrisch”, “sleeve”, and “Magenslauch”. Eligible cases that met inclusion criteria were further divided depending on *Hp*-Status into positive and negative ones. Retrieved results from the previously described procedure were controlled independently by two investigators (M.D. and N.T.P.), according to the inclusion and exclusion criteria. Selected data were validated by D.S.S. In cases of conflict, a consensus was met by the intervention of a senior author (A.K.Ε). The following parameters were available after extraction from “E.care” software to Excel sheet for statistical analysis: (a) demographics and anthropometric parameters (age, gender, body mass index [BMI]), date of admission and discharge; patients’ countries of origin were coded and grouped as defined by the United Nations (Standard country or area codes for statistical use, M49)) [31]; (b) active smoking status; (c) main histological findings of gastric mucosa biopsies; (d) MetS parameters including arterial hypertension, dyslipidemia, IR and T2DM, as stated by the Expert Committee on the Diagnosis and Classification of Diabetes [32]. Dyslipidemia was defined once levels of triglycerides exceeded 150 mg/dL (1.7 mmol/L) and/or LDL-C levels 100–160 mg/dL (2.58–4.13 mmol/L, depending on other risk factors) and/or HDL-C levels were lower than 40 and 50 mg/dL (1.03 and 1.29 mmol/L) for men and women, respectively, or treatment with hypolipidemic medication(s). Arterial hypertension was defined as systolic blood pressure ≥ 130 mmHg and/or diastolic blood pressure ≥ 85 mm Hg or treatment with antihypertensive drugs). T2DM and IR were defined by serological tests including fasting glucose, fasting insulin (for the estimation of IR, using the homeostasis model assessment–IR method (HOMA–IR) [33]), and glycated hemoglobin A1c (Hba1c).

### 2.5. Statistical Analysis

Both uni- and multivariate logistic regression analyses were performed with the binary outcome presence/absence of *Hp*-I in the gastric mucosa biopsy and the following variables: age, gender, nationality (Swiss vs. other, Western Europe vs. other, Balkans vs. other), dyslipidemia, arterial hypertension, GA and IM, active smoker status and T2DM. HOMA–IR between *Hp*-I groups was compared by means of Student’s *t*-test. To select the variable included in the multivariate analysis, we used a backward elimination procedure (or all significant variables in the univariate analysis were considered for the multivariate analysis). Values are presented as the mean (±standard deviation) or median (range) for normally and non-normally distributed, respectively, continuous variables. A two-tailed *p* value < 0.05 was considered statistically significant. Missing values were ≤ 5% in the data set, and no imputation strategies were used. All calculations were conducted using R Project for Statistical Computing (The R Foundation, Version 3.2.0, Vienna, Austria).

## 3. Results

A total of 116 patients met the inclusion criteria and were recruited for the statistical analysis. Of those, 39 were male, whereas the remaining 77 (66.4%) female individuals. The mean age of all patients was 48.66 (±12.55) years. The main descriptive statistics are enclosed in Table 1.

Gastric mucosa biopsies with *Hp*(+) were found in 28/116 (24%) of the patients. Of the *Hp*(+) patients, 10 were males (36%). There was no statistically significant difference between *Hp*(+) and *Hp*(−) groups in terms of age, BMI, and gender. Presence of GA was documented in 8/28 *Hp*(+) patients (29%) (6.8% of total population) and 2/88 *Hp*(−) ones (2%, *p* = 0.006).

Likewise, IM was demonstrated in 14/28 (50%) patients of *Hp*(+) group (12% of total population) versus 3/88 (3.4%) of the negative one (*p* < 0.0001). Moreover, *Hp*(+) patients exhibited significantly increased arterial hypertension (*p* = 0.0332). T2DM, dyslipidemia, triglycerides, active smoker status did not differ significantly between groups. Subgroup analyses between different geographical origins of patients did not yield any statistical significance either in terms of *Hp* status (Table 2).

**Figure 1:**
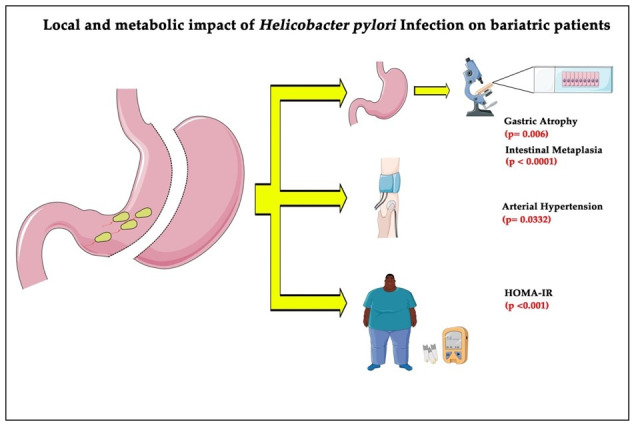
Patients undergoing bariatric surgery were significantly associated with gastric atrophy, intestinal metaplasia, arterial hypertension, and increased insulin resistance when infected with *Hp*, compared to the noninfected patients. HOMA–IR, homeostasis model assessment–insulin resistance method.

No patients were found with dysplasia of gastric mucosa. Considering the HOMA–IR score (Figure 1), an independent Student’s *t*-test comparison of means between *Hp*(+) and *Hp*(−) revealed a statistical significance (*p* < 0.001, difference of means −4.003 ±0.980). In the multivariate analysis, which included arterial hypertension, GA, and IM as independent variables, statistical significance remained only for the IM (*p* = 0.001 but not for GA (*p* = 0.074) and arterial hypertension (*p* = 0.467).

## 4. Discussion

To our knowledge, this series showed, for the first time in Switzerland, that *Hp*(+) individuals exhibit significantly more frequently certain MetS parameters with concomitant premalignant histological lesions including GA and IM without dysplasia, compared to their *Hp*(−) counterparts. Moreover, we demonstrated a *Hp*-I prevalence for Switzerland of about 24%, which is in line with previous studies of us [14] and others [34] reporting a prevalence of around 20%. Nevertheless, early studies by others displayed discrepant results with a Swiss patient *Hp* prevalence as high as 73% [35] or as low as 9% [36], thereby requiring further investigation. Differences in methodology and/or inclusion of different genetic populations might explain this discrepancy.

Regarding our own histological premalignant findings, compatible results were reported by a large-scale Italian study [4]; the authors examined the histopathological specimens of 474 morbidly obese patients undergoing laparoscopic gastric sleeve surgery and equally observed a statistically significant presence of premalignant lesions including GA, IM, or dysplasia for the *Hp* positive patients. However, the reported presence of IM (4%) and GA (2.7%) was lower. Moreover, the authors did not consider concomitant MetS parameters. A relevant study of equal power was conducted in Lebanon [18], where the authors reported a higher *Hp* prevalence among bariatric patients (35.3%) and a lower IM rate (1.7%), again without evaluation of MetS components. On the other hand, an additional recent gastric sleeve database from Egypt [37] investigating the relationship among *Hp*, sleeve surgery, and gastroesophageal reflux, in a total of 176 specimens being reviewed, found a higher *Hp* prevalence (39%) without GA, IM, or association with DM, hyperlipidemia, and arterial hypertension. Again, differences in the origin of studies performed, methodology, or interpretation of findings might explain, at least partly, the aforementioned contradictory results.

Important to note, however, is that apart from the well-established local precarcinogenic virulence of *Hp*-I on gastric mucosa, as it was firstly proposed by the Correa-cascade [9], *Hp* also displays a strong association with MetS parameters that, beyond other conditions, contribute to gastric pathologies. Nevertheless, it has to be acknowledged that association never means causation since Bradford–Hill criteria have to be satisfied [38]. Moreover, the plethora of emerging evidence being accumulated justifies as reasonable to propose pathogenetic hypotheses of the *Hp*-I effect. In this regard, an Egyptian study recruited 99 patients with arterial hypertension and *Hp*-I. After eradicating *Hp*, blood pressure was normalized in the majority of patients (91%), and they subsequently quitted their antihypertensive medication [39]. Furthermore, our series showed that the *Hp*-I was associated with arterial hypertension, as one of the MetS components. Additional studies focusing on non-bariatric populations further reinforce our findings; as early as 2003, an Italian study showed that *Hp* eradication had a beneficial effect on arterial hypertension, and the authors postulated pathogenetically a cytokine cascade activation with the subsequent production of vasoactive substances from the stomach or molecular mimicry between peptides expressed by smooth muscle, as well as endothelial cells and the CagA antigen of *Hp* [40]. Supportive results were also reported by a large-scale Chinese study (*n* = 5246), in which *Hp*(+) individuals were characterized by an increased prevalence of arterial hypertension [41]. Likewise, recent data indicate that, beyond other parameters, *Hp*-I and arterial hypertension are significantly related to gastric premalignant lesions [42]. Moreover, patients with arterial hypertension display an increased risk of GC development; arterial hypertension is associated with a twofold increased risk of gastric cardia adenocarcinoma [43]. Specifically, GC and arterial hypertension appear to share a biochemical pathway of augmented concentrations of inositol triphosphate and cytosolic calcium that may contribute to the pathogenesis of arterial hypertension and gastric oncogenesis [44].

Potential mechanisms involved in the pathophysiology of *Hp*-related arterial hypertension appear to include the following:

(a) High-salt diet, a known risk factor for arterial hypertension, which favors *Hp* colonization [45];
(b) *Hp*-mediated inflammatory processes that have been associated with atherosclerosis [46] and *Hp*-induced inflammatory cytokines involved in the pathophysiology of arterial hypertension [47];
(c) *Hp*-associated MetS parameters, such as *Hp*-related IR, the major underlying mechanism responsible for MetS [48], which also plays an important role in the pathogenesis and progression of arterial hypertension-triggered target organ injury [49].

However, further research is warranted to elucidate in depth the mechanisms involved in *Hp*-related arterial hypertension, a serious clinical disorder with high worldwide incidence and prevalence that continues to increase and contributes to global morbidity and mortality [50].

Apart from arterial hypertension, the present series also showed that the *Hp*-I was associated with a statistically relevant IR, the key pathogenic mechanism underlying MetS, which promotes the oncogenesis of diverse malignancies, including GC [48,51]. As early as 2011, we reported an association between *Hp*-I and IR within a systematic review [52]. Moreover, in a further study, we demonstrated, by obtaining liver and stomach specimens for characterization of NAFLD and *Hp*-I, respectively, that *Hp*(+) bariatric patients were characterized by higher MetS components (including HOMA–IR, arterial hypertension, and dyslipidemia) than the negative ones [14]. It is of note that NAFLD is associated with an increased risk of malignancies including GC, whereas bariatric intervention offers a significant decrease in the risks of any malignancy and obesity-related cancer in severely obese NAFLD patients [53]. Comparable results in terms of IR and *Hp*-I association were exhibited in further studies in the past [54,55]. For instance, Chen et al. [56] demonstrated, in a Taiwan population, that *Hp*(+) patients were characterized by a higher HOMA–IR than their *Hp*-negative counterparts, and for patients younger than 50 years of age, *Hp* served as a predictor for MetS. Meta-analyses connected *Hp*-I with glycemic control and diabetes complications [57]. In this regard, T2DM, an essential component of MetS associated with *Hp*-I [58,59], is considered a risk factor for GC [60], and the potential relationship between T2DM and GC has been considered for several years owing to their common characteristics, including hyperinsulinemia, hyperglycemia, and inflammation [61]. Specifically, there are several proposed mechanisms to clarify the pathogenic role of T2DM in gastric oncogenesis. Patients with hyperglycemia could develop IR, and hyperinsulinemia may cause cell proliferation. This process could induce alterations in the gastric mucosa and genetic changes, ultimately resulting in gastric oncogenesis [62]. Likewise, proinflammatory cytokines such as interleukin (IL)-6, are related to IR in patients with malignancies including GC [63]; proinflammatory cytokines, such IL-6, have been related with inflammatory responses, which contribute to carcinogenesis [64]; the chronic inflammatory process may raise the incidence and mortality of malignancies in patients with T2DM [65]; and, both T2DM and *Hp*-I increase the risk of GC, accompanied by more severe gastric inflammatory process [66]. In addition, rises in levels of reactive oxygen species induced by T2DM are connected with mutational alterations in oncogenes and tumor suppressor genes. This process contributes to gastric oncogenesis [67]. Furthermore, the rise in insulin-like growth factors in patients with T2DM plays an essential role in the initiation, progression, and metastases of GC [67].

In line with our findings, relative data indicate that Mets-related *Hp*-I, dyslipidemia, arterial hypertension, T2DM, smoking, alcohol consumption, diet (salty and/or spicy diets), and even sarcopenia appear to be underlined denominators significantly associated with precancerous gastric mucosa lesions including GA, IM, and dysplasia [42]. These precancerous lesions progress to GC because of several influencing factors [9,68]. In this respect, for instance, *Hp*-I and T2DM display synergistic effects on gastric carcinogenesis [69]. Since the chronic inflammatory process plays a role in the development of AG and GC [70], inflammation has also been involved as an essential etiologic factor in both IR and T2DM, and a higher proportion of AG (43.1 vs. 24.6%, *p* < 0.001) and IM (13.7 vs. 7.1%, *p* < 0.001) are observed in patients with T2DM than in those without T2DM [71]. Likewise, hyperglycemic patients could develop IR, and increased insulin levels may induce cell proliferation. This process may provoke changes in the gastric mucosa and genetic alterations, ultimately resulting in gastric oncogenesis [62]. Moreover, T2DM-induced increased levels of reactive oxygen species and hyperglycemia are linked with mutational alterations in oncogenes and tumor suppressor genes, thereby contributing to gastric oncogenesis [67,72]. Furthermore, the rise in insulin-like growth factors in T2DM seems to play a significant role in the initiation via premalignant lesions, progression, and metastasis of GC [73,74]. Regarding the relationship between hypertension and GC, again, for instance, increased concentrations of inositol triphosphate and cytosolic calcium appear to be involved in the pathogenesis of hypertension and GC stage [44].

Another interesting observation of our study, albeit not reaching statistical significance, is gender tropism. The participation of women was as high as 66.4% (77/116) and 64% for the *Hp*-I ones (18/28). This observation is in line with previous bariatric studies reporting a predominance of women undergoing bariatric surgery [75,76]. Additionally, *Hp*-I is known in the literature to affect more women than men [77,78], although studies reporting a male predominance ought to be equally acknowledged [79]. Furthermore, estradiol-treated gerbils exhibited more intense and extended acute and follicular gastritis, compared to the control group [80]. On the contrary, the male gender, compared to premenopausal women, is regarded for MetS with its hepatic component (i.e., NAFLD) as an independent risk factor, since estrogens are believed to be likely beneficial for premenopausal women [2,81].

Beyond our findings, other relative data also indicate that *Hp*-I is linked with MetS, particularly in females; the MetS prevalence is higher in *Hp*-infected women than in men (women: 7.4% vs. 2.5%, *p* < 0.001) [82]. Some possible insights include the following: females appear to exhibit a higher *Hp* burden than males [83]. Likewise, *Hp*(+) females display significantly decreased HDL cholesterol concentrations than males, whereas total cholesterol, LDL cholesterol, and triglycerides are statistically higher in *Hp*(+) males, thereby signifying that *Hp* might affect lipid profiles and may be different by gender [78]. Moreover, the mentioned T2DM, a component of MetS, and *Hp*-I have synergistic effects on gastric oncogenesis [69]. In this respect, hyperglycemia and high composite MetS score are connected with an increased risk of gastric cancer (GC) only in females; positive associations are detected for females but not in males with high waist circumference, arterial hypertension, high fasting, and nonfasting glucose and GC [60,84]. Finally, *Hp* eradication regimens are significantly unsuccessful in females than in males. Gender differences in acid output and gastric blood flow have been suggested to influence eradication treatment outcomes [85]. Nevertheless, the mechanism(s) that underlie these differences demand further investigation.

Important to note is that the epidemic of obesity and the MetS are essential causes of morbidity and mortality, and metabolically abnormal obese people appear to exhibit a higher risk for incident GC than metabolically healthy obese ones [86]. In this respect, there is a potential impact of *Hp*-associated MetS on the sequence chronic active gastritis-GA—IM—dysplasia-gastric oncogenesis [48].

An attractive further pathogenic feature of Hp consists of the various associated virulence factors which Hp may express; a plethora of molecules, the major of which are vacuolating cytotoxin A (VacA), cytotoxin associated gene A (CagA), and α-subunit of urease (UreA) compose this capacity. Strains expressing these virulence factors are known to exhibit enhanced pathogenicity. Further distinct virulence genes expressed by *Hp* are SabA, OipA, BabA, and HopQ, which play a significant role in bacterial colonization [87]. The most studied of all is CagA, which is considered also a bacterial oncoprotein, due to the oncogenic pathways in which it is implicated. [88,89]. Moreover, CagA, which is found in about 50% of Hp strains, is regarded as a key player in the cardiovascular involvement of Hp; formation of cholesterol plaques in arterial walls and induction of autoimmunity are attributed, at least partly, to CagA [90]. Additionally, CagA-positive Hp strains have been associated previously with higher values of Hba1c [91].

Similarly, we were not able to evaluate lifestyle and especially diet in our population; in a Chinese study (n = 3014), it was revealed that a rich-in-salt diet is linked with Hp-I, whereas a “vegetable and grain” dietary pattern is associated with a diminished risk of Hp-I [92]. Additionally, in a recent study focusing on the same ethnic population (*n* = 182), it was demonstrated that *Hp*-I (+) individuals following a high-fiber rye diet had a lower high sensitive C-reactive protein, as well as low-density lipoprotein cholesterol, compared to a control group following a refined-wheat diet [93]. A third large scale (n = 10,407) Chinese study deduced that a carbohydrates/sweets-based diet was positively associated with the Hp-I, whereas a high intake of animal offal, animal blood, fish, seafood, and poultry diet was inversely associated with Hp-I prevalence [94].

This study has some limitations, the main of which include the retrospective nature of the design as well as the small sample size. The latter was found, however, adequate to yield a statistical significance for the above-mentioned key components of MetS and gastric oncogenesis. Moreover, due to the mentioned retrospective design as well as ethical approval-related restrictions, we were unable to identify the *Hp* strains, which mediated the local and metabolic virulence to our studied population. A further limitation is the inability to collect information regarding nutrition and general lifestyle due to the particularity of the ED setting.

## 5. Conclusions

This Swiss pilot study showed that *Hp*-I is associated with both histologic premalignant lesions, including GA and IM, and MetS components, i.e., arterial hypertension and IR. Further large-scale prospective studies are warranted in order to reinforce the presented scientific evidence. Mechanistical (preclinical) studies are also needed so as the complex pathogenetical pathways of *Hp* pleotropic virulence are unraveled.

## Figures and Tables

**Table 1:** Descriptive statistics.

Parameter	Minimum	Maximum	Mean	SD
Age (years)	24	75	48.66	12.55
BMI (kg/m^2^)	30.7	76	54.10	8.68
HOMA-IR	0.2	30	5.41	4.58
Hba1c (%)	4.2	10.8	5.97	1.12
**Parameter**	**Value**	**Number**	**Percentage**	
Gender	Female	77	66.4%	
	Male	39	33.6%	
Origin	South America	3	2.6%	
	Southern Asia	1	0.9%	
	Southern Europe	12	10.3%	
	Western Asia	4	3.4%	
	Eastern Europe	6	5.2%	
	Northern Europe	3	2.6%	
	Western Europe	87	75%	
*Hp* Status	positive	28	24.1%	
	negative	88	75.9%	
Dyslipidemia	present	62	54.4%	
	absent	52	45.6%	
Triglycerides	present	32	31.1%	
	absent	71	68.9%	
(Pre)diabetes	present	61	52.6%	
	absent	55	47.4%	
Arterial hypertension	present	58	50%	
	absent	58	50%	
Active smoker	positive	22	20.4%	
	negative	86	79.6%	
Intestinal metaplasia	present	17	14.7%	
	absent	99	85.3%	
Gastric atrophy	present	10	8.6%	
	absent	106	91.4%	

BMI; body mass index, Hba1c; glycated hemoglobin, HOMA–IR; homeostasis model assessment–insulin resistance; *Hp*; *Helicobacter pylori*, SD; standard deviation.

**Table 2:** Comparison of different variables between *Helicobacter pylori* positive and negative groups.

Variable	OR (95% CI)	*p*-Value	Description
Gender	1.13 (0.45;2.72)	0.788	Man/women
Swiss Nationality	0.58 (0.24;1.44)	0.23	Swiss vs. other Nation
Western Europe	0.45 (0.18; 1.14)	0.088	Western EU vs. other
Balkans	1.67 (0.42; 5.79)	0.436	Balkans vs. other
Dyslipidemia	1.59 (0.66; 3.96)	0.308	Yes/no
Age	1.002 (0.99; 1.008)	0.509	Continuous
Diabetes	0.966 (0.995; 1.008)	0.93	(Pre)diabetes vs. normal
Triglycerides	1.058 (0.386; 2.735)	0.908	High/normal
Active smoker	0.521 (0.114; 1.733)	0.332	Yes/no
Arterial hypertension	2.65 (1.105; 6.766)	0.0332 *	Yes/no
Intestinal metaplasia	28.3 (8.07; 135.0)	<0.0001 *	Yes/no
Gastric atrophy	17.2 (3.95; 119.9)	<0.001 *	Yes/no

* Statistical significance, CI: confidence interval, OR: Odds Ratio.

## Data Availability

Data sharing not applicable.
